# Antifungal Nanoparticles in Microbial Biotechnology: Multisector Mechanisms, Applications, and Future Frontiers

**DOI:** 10.1049/nbt2/7866252

**Published:** 2026-05-30

**Authors:** Kaveh Rahimi Mamaghani, Marzieh Alikarami, Hossein Saremi, Nader Parvin

**Affiliations:** ^1^ Department of Materials and Metallurgical Engineering, Amirkabir University of Technology (Tehran Polytechnic), Tehran, Iran, aut.ac.ir; ^2^ Department of Plant Protection, College of Agriculture and Natural Resources, University of Tehran, Karaj, Iran, ut.ac.ir

**Keywords:** agricultural nanotechnology, antifungal nanoparticles, biofilms, microbial biotechnology, nanobiofungicides, safe-by-design nanomaterials, soil microbiome, *Trichoderma*

## Abstract

Fungal pathogens threaten global food security, ecosystem stability, and human health, while the overuse of conventional fungicides accelerates resistance and disrupts beneficial microbiota. Nanotechnology offers a powerful new paradigm for fungal control, but its greatest potential lies at the interface with microbial biotechnology. Here, we critically review advances in antifungal nanoparticles (NPs) with a focus on their integration into microbiome‐aware and microbe‐assisted systems. We discuss biogenic NP synthesis by bacteria, fungi, and yeast; synergistic nanobiofungicides that combine NPs with biocontrol agents such as *Trichoderma* and *Bacillus*; and NP‐enabled strategies that selectively suppress pathogens while preserving beneficial taxa. Mechanistic insights include NP‐mediated membrane disruption, reactive oxygen species (ROS) generation, ion release, and biofilm inhibition, with special attention to how these processes modulate plant‐microbe interactions in the rhizosphere. Translational applications span pre‐ and postharvest agriculture, seed treatments, microbial inoculant stabilization, and smart delivery systems for agrochemicals. We also examine the ecological implications of NP deployment, highlighting safe‐by‐design (SbD) strategies, biodegradability, and microbiome resilience as key design criteria. Finally, we outline a future roadmap where nanotechnology converges with synthetic biology, microbial engineering, and AI‐guided design to enable precision antifungal systems that are adaptive, ecologically compatible, and scalable. Together, these insights position antifungal nanotechnology as a next‐generation tool in microbial biotechnology, with the potential to reshape crop protection, soil health management, and sustainable fungal control.

## 1. Introduction: The Fungal Challenge and the Nanobiotechnology Response

Fungal diseases represent a rapidly escalating threat to human health, food systems, ecosystems, and infrastructure worldwide [1]. Once considered secondary to bacterial and viral infections, fungal pathogens are now recognized as among the most difficult microbial threats to control. This is largely due to their eukaryotic nature, capacity for drug resistance, and ability to persist in diverse environmental niches [2]. Globally, fungi are estimated to destroy 10%–23% of crop yields preharvest and an additional 10%–20% postharvest, accounting for more than $60 billion in annual losses in staple food production alone [3]. At the same time, invasive fungal infections cause an estimated 1.6 million human deaths per year, surpassing malaria and rivaling tuberculosis in global mortality [4, 5]. Despite this growing threat, the arsenal of antifungal agents remains limited and technologically stagnant. In both clinical and agricultural contexts, antifungal therapies remain constrained by reliance on a limited number of chemical classes, primarily azoles, echinocandins, and polyenes. These agents suffer from poor solubility, limited bioavailability, host toxicity, and the continual emergence of resistance [6]. In agriculture, overuse of site‐specific fungicides such as strobilurins and triazoles has accelerated multidrug resistance (MDR) in major phytopathogens, including *Fusarium*, *Botrytis*, *Alternaria*, and *Magnaporthe* species [7]. Clinically, azole resistance in *Candida*, particularly *Candida auris*, has become a major concern in hospitals and intensive care units, where mortality rates remain high despite antifungal intervention [8, 9].

To address these challenges, nanotechnology has emerged as a versatile tool in antifungal innovation [10]. Nanoparticles (NPs), broadly defined as particles with at least one dimension below 100 nm, provide multifunctional platforms to enhance antifungal activity through intrinsic toxicity, drug delivery, biofilm penetration, and synergy with existing compounds [11]. Inorganic NPs such as silver, copper, and zinc oxide exhibit potent fungicidal activity via membrane disruption, ion release, and oxidative stress, with pleiotropic mechanisms that reduce the likelihood of resistance evolution [12, 13]. Organic or hybrid nanocarriers, including liposomes, polymeric systems, and silica shells, can solubilize poorly water‐soluble antifungals, improve tissue targeting, and enable sustained or stimuli‐responsive release [14]. NPs can also disrupt biofilm matrices, inhibit spore germination, and directly interfere with hyphal development [15]. Additionally, green or biogenic synthesis methods using plant extracts, microbial metabolites, or biodegradable polymers allow for safe‐by‐design (SbD) nanomaterials that maximize efficacy while minimizing harm to nontarget organisms and the environment [16, 17]. From a microbial biotechnology perspective, NPs are increasingly integrated into microbiome‐aware agricultural systems. In these systems, nanoagents selectively suppress pathogens while preserving beneficial soil microbes [18]. Recent developments also include nanobiofungicides that couple NPs with biocontrol microbes such as *Trichoderma* and *Bacillus* species. These systems leverage both biological and nanoscale mechanisms to support sustainable plant disease management [19, 20]. Furthermore, NPs offer scalable solutions for reducing postharvest spoilage, a major contributor to global food waste, through applications such as packaging films embedded with antifungal nanomaterials that extend the shelf life of perishable produce [21, 22].

Several recent reviews have examined antifungal NPs from specific angles; for example, Zhu et al. [23] provide an extensive exploration of nanomaterials for treating fungal infections from a primarily biomedical perspective, focusing on overcoming drug resistance. Other works have summarized mechanisms and material properties of metal‐based antifungal NPs without integrating broader ecological or regulatory context [24], and some highlight agricultural applications of green NPs for crop protection without addressing microbial ecosystems or SbD frameworks [25]. In contrast, the present review systematically integrates mechanistic insights with microbiome‐aware design, nanobiofungicides, and regulatory challenges in microbial biotechnology, thereby presenting a holistic and application‐oriented perspective that spans agriculture, food safety, and biomedicine (Supporting Information: Table S1). In other words, unlike previous reviews that focused mainly on material chemistry or clinical aspects, this work specifically integrates antifungal nanotechnology with microbial biotechnology, highlighting nanobiofungicides, soil‐microbiome interactions, and SbD strategies as emerging frontiers. Despite these advances, significant limitations persist. Potential toxicity to nontarget organisms, environmental accumulation, inconsistent NP behavior across biological systems, and fragmented regulatory frameworks continue to hinder the widespread adoption of antifungal nanomaterials [26, 27]. Consequently, this review aims to critically evaluate the current landscape of antifungal NPs with a special emphasis on their applications in agriculture, food systems, and microbial biotechnology. It will examine NP classification and design, mechanisms of fungal inhibition at the nanobio interface, applications in pre‐ and postharvest contexts, interactions with microbiomes and ecosystems, safety and regulatory considerations, and future trends in smart nanocarriers and sustainable deployment.

## 2. Classification of Antifungal NPs

**Figure 1:**
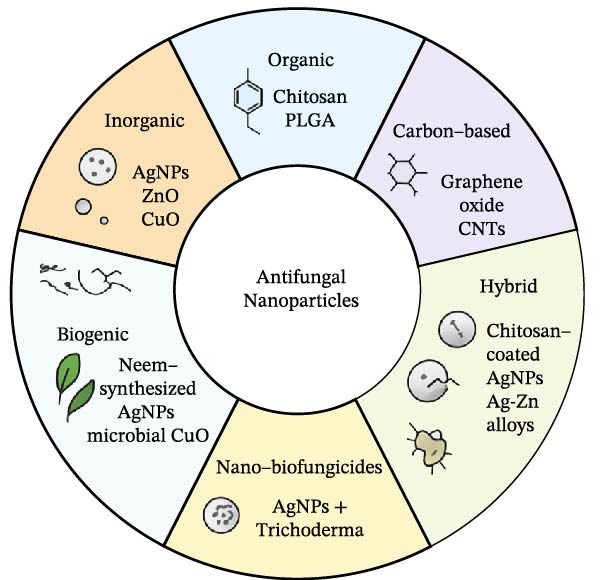
Classification of antifungal NPs into six major categories: (1) inorganic (metals/oxides), (2) organic (polymers/lipids), (3) carbon‐based nanomaterials, (4) hybrid/composite NPs, (5) biogenic/green‐synthesized NPs, and (6) nanobiofungicides combining NPs with microbial biocontrol agents. This schematic is a conceptual framework classification from reported literature trends.

Antifungal NPs encompass a broad range of nanoscale materials designed to inhibit fungal growth through direct or indirect mechanisms, and their classification is typically based on core composition, surface functionality, synthesis method, and application domain [28]. For microbial biotechnology and agricultural deployment, a practical taxonomy must consider both physicochemical diversity and biological behavior within complex environments such as the rhizosphere, phyllosphere, and postharvest surfaces [29]. Based on these criteria, antifungal NPs can be grouped into six major categories: inorganic NPs (metals and metal oxides), organic NPs (polymers and lipids), carbon‐based nanomaterials, hybrid and composite nanostructures, biogenic or green‐synthesized NPs, and nanobiofungicides that combine NPs with microbial biocontrol agents. The classification of antifungal NPs can be visually summarized in Figure 1, which outlines the six major categories based on core composition and functional integration.

### 2.1. Inorganic NPs (Metals and Metal Oxides)

Inorganic NPs, particularly those composed of metals and metal oxides, are the most extensively studied due to their broad‐spectrum antifungal activity. Silver NPs (AgNPs) are among the most prominent, exhibiting fungicidal activity against species such as *Candida*, *Aspergillus*, *Botrytis*, and *Fusarium* through mechanisms involving Ag^+^ ion release and reactive oxygen species (ROS) production [30, 31]. Their utility extends to agricultural formulations in foliar sprays, seed coatings, edible films, and smart packaging materials [32]. Copper‐based NPs, including Cu and CuO forms, are valued for their cost‐effectiveness and strong efficacy against phytopathogens such as *Phytophthora infestans*, *Alternaria solani*, and *Colletotrichum* spp., acting primarily through ion release and oxidative stress pathways [33]. Zinc oxide NPs (ZnO NPs), notable for their photocatalytic and dark‐activated antifungal activity, inhibit conidial germination, disrupt hyphal membranes, and generate ROS, and they are widely used in postharvest coatings and nanoenabled fertilizers that simultaneously deliver antimicrobial and nutritional benefits [34, 35]. Titanium dioxide NPs (TiO_2_ NPs) leverage photocatalytic ROS generation, making them useful in antimicrobial paints, greenhouse films, and water treatment systems, though their activity is strongly dependent on crystal phase and light exposure [36]. Iron oxide NPs (Fe_2_O_3_ or Fe_3_O_4_) are less potent as standalone fungicides but serve as magnetic carriers for drug delivery and hybrid systems due to their low toxicity and soil enrichment potential [37]. Other metal‐based nanostructures, including gold, magnesium oxide, selenium, and platinum‐group NPs, are also under investigation; while AuNPs are often explored as delivery vehicles, selenium NPs (SeNPs) have shown particular promise in disrupting biofilms and enhancing crop growth [38, 39]. The antifungal efficacy of inorganic NPs is strongly influenced by physicochemical parameters such as size (with particles smaller than 20 nm often being more potent), shape, crystallinity, and surface charge. However, higher reactivity may also result in phytotoxicity or negative impacts on beneficial microbes, which necessitates surface modifications such as PEGylation or polymer capping to balance biocompatibility with antifungal potency.

### 2.2. Organic NPs (Polymers and Lipids)

Organic NPs, including polymeric, lipid, and protein‐based carriers, are primarily used to encapsulate antifungal agents, enhance solubility, and enable targeted or controlled delivery. Polymeric NPs such as PLGA, PLA, PCL, and chitosan‐based systems can deliver synthetic or natural antifungals, with chitosan NPs providing inherent antifungal activity and stimulating plant defense responses [40]. Lipid‐based NPs, including liposomes, nanoemulsions, and solid lipid NPs, enhance the solubility of poorly water‐soluble antifungals such as itraconazole and amphotericin B, facilitating their oral or topical use in medicine and agriculture [41]. Protein‐based nanocarriers, such as bovine serum albumin NPs, offer biodegradable delivery systems that stabilize antifungals and provide opportunities for multifunctional formulations [42]. While organic NPs are generally less toxic and more biodegradable than their inorganic counterparts, they typically lack intrinsic antifungal activity and are therefore most effective as carriers rather than direct fungicides.

### 2.3. Carbon‐Based Nanomaterials

Carbon‐based nanomaterials provide unique antifungal mechanisms due to their structural features and high surface area. Graphene oxide (GO) sheets exhibit mechanical membrane disruption and ROS‐mediated activity and are often hybridized with silver or zinc oxide for enhanced efficacy [43]. Carbon nanotubes (CNTs) can physically pierce fungal membranes and act as scaffolds for antifungal drug loading, though their aggregation and persistence raise safety concerns [44]. Carbon dots (CDs), by contrast, are small fluorescent nanodots with promising antifungal and bioimaging potential due to their functionalization versatility [45]. Fullerenes (C60) are less common but are under study for photodynamic fungal inactivation [46]. Carbon nanostructures are often incorporated into hybrid systems with metals or polymers to improve dispersibility and reduce toxicity; however, environmental persistence remains a regulatory concern.

### 2.4. Hybrid and Composite NPs

Hybrid and composite NPs are designed to leverage multiple antifungal mechanisms through structural integration. Examples include polymer‐coated metal NPs such as chitosan‐coated AgNPs, which combine ion release with plant immunity stimulation, and bimetallic systems such as Ag‐Cu or Ag‐Zn alloys that broaden antifungal spectrum and reduce resistance evolution [47]. Core‐shell nanostructures such as Ag cores with silica shells provide controlled ion release and enhanced environmental stability, while magnetic nanocomposites enable targeted delivery and recovery after application [48]. Smart composites, engineered to release antifungals in response to environmental cues such as pH shifts or fungal enzyme activity, represent the frontier of responsive antifungal systems [49]. While these hybrid platforms offer modularity and tunability, their complex synthesis and regulatory challenges may limit large‐scale adoption.

### 2.5. Biogenic and Green‐Synthesized NPs

Biogenic and green‐synthesized NPs are increasingly central to sustainable nanotechnology efforts. Using plant extracts, microbial metabolites, or fungal systems for NP synthesis produces particles with natural surface coronas that improve stability and biocompatibility [50]. Plant‐based synthesis methods using extracts of neem, lemongrass, aloe, or green tea have yielded Ag, ZnO, and CuO NPs with high antifungal activity against *Fusarium*, *Alternaria*, and *Botrytis* species [51]. Recent studies confirm that yeast species such as *Yarrowia lipolytica* can biosynthesize SeNPs with potent biological activity [52], underlining the promise of microbial factories for eco‐friendly antifungal NP production. Microbial synthesis using *Trichoderma*, *Aspergillus*, *Bacillus*, or *Pseudomonas* spp. has generated bio‐NPs with strong compatibility and antifungal potential, bridging microbial biotechnology with nanoscience [53]. Such eco‐friendly strategies align closely with principles of regenerative agriculture and provide cost‐effective and scalable production routes.

### 2.6. Nanobiofungicides (NPs + Microbial Biocontrol Agents)

A particularly promising category is nanobiofungicides, which integrate antifungal NPs with living microbial antagonists such as *Trichoderma*, *Bacillus*, or *Pseudomonas* [54]. These hybrid systems enhance pathogen suppression via synergistic antifungal mechanisms, stabilize beneficial microbes on seeds or soil particles, codeliver metabolites and ions, and extend field persistence. Reported examples include AgNPs synthesized using *Trichoderma harzianum* and applied to Fusarium‐infected soils, chitosan NPs loaded with *Bacillus* metabolites for root treatment, and mesoporous silica NPs coloaded with spores and essential oils [55]. Such systems exemplify the goals of microbial biotechnology by coupling nanoscale mechanisms with ecological precision, offering sustainable alternatives to synthetic fungicides. These hybrid NP‐microbe systems epitomize the principles of microbial biotechnology, where engineered or natural microbes act not just as NP synthesizers but as cofunctional partners in sustainable disease suppression.

In summary, antifungal NPs can be classified into six major categories based on their material composition and functional integration. Each class offers unique advantages and limitations in terms of antifungal potency, environmental safety, and scalability, highlighting the need for tailored design strategies that balance efficacy with ecological compatibility. Table 1 summarizes the primary categories of antifungal NPs along with representative examples, synthesis strategies, antifungal mechanisms, and their relevance to agricultural applications.

**Table 1:** Summary of NP categories, key examples, synthesis routes, antifungal mechanisms, and agricultural relevance.

NP category	Key examples	Synthesis routes	Antifungal mechanisms	Agricultural relevance	Key refs.
Metallic NPs	Silver (Ag), copper (Cu), zinc (Zn)	Green synthesis (plant extracts), chemical reduction, sol‐gel, hydrothermal	ROS generation, disruption of fungal membranes, protein inactivation, ion release	Widely tested against phytopathogens; potential for crop protection formulations	[31, 56]
Metal oxide NPs	ZnO, TiO_2_, CuO, MgO	Precipitation, thermal decomposition, biosynthesis	Membrane damage, photocatalytic ROS production, enzyme inhibition	UV‐activated antifungal sprays, postharvest disease control	[35, 57, 58]
Carbon‐based NPs	Graphene oxide (GO), carbon nanotubes (CNTs), fullerenes	Exfoliation, CVD, ball milling, microbial synthesis	Physical piercing of membranes, oxidative stress, interference with cellular respiration	Emerging use in antifungal coatings, seed priming	[59, 60]
Polymeric NPs	Chitosan, PLGA, PEGylated systems	Ionic gelation, emulsion polymerization, nanoprecipitation	Disruption of fungal cell walls, sustained antifungal release, immunomodulation	Biodegradable and safe, potential seed coatings and foliar delivery	[61, 62]
Lipid‐based NPs	Solid lipid NPs, liposomes, nanostructured lipid carriers (NLCs)	High‐pressure homogenization, solvent evaporation, microemulsion	Encapsulation of antifungal agents, enhanced delivery and penetration into fungal cells	Promising carriers for fungicides and biopesticides, reduced toxicity	[63]
Silica‐based NPs	Mesoporous silica nanoparticles, SiO_2_	Sol‐gel synthesis, templated growth	Controlled release of antifungal agents, ROS production	Soil amendments, smart delivery vehicles for agrochemicals	[64, 65]
Hybrid/composite NPs	Ag‐chitosan, ZnO‐graphene, polymer‐metal hybrids	Combination of green and chemical synthesis	Synergistic multimechanistic antifungal effects	Designed for multifunctional antifungal coatings and crop protection formulations	[59, 66]

## 3. Mechanisms of Antifungal Action

The NPs exert antifungal effects through a multifaceted combination of physical, chemical, and biological mechanisms. Unlike conventional fungicides, which usually target a single enzyme or receptor, such as lanosterol demethylase in the case of azoles, NPs interact with multiple cellular structures and processes simultaneously. This multihit strategy not only enhances their efficacy but also decreases the likelihood of resistance development, making them especially promising against MDR and biofilm‐forming pathogens [67]. The antifungal effects of NPs occur at various biological levels, from spore germination and surface adhesion to intracellular stress responses and gene regulation, and are influenced by NP size, morphology, surface charge, composition, functionalization, and the surrounding biological environment [68–70].

### 3.1. Disruption of Cell Membrane and Wall Integrity

**Figure 2:**
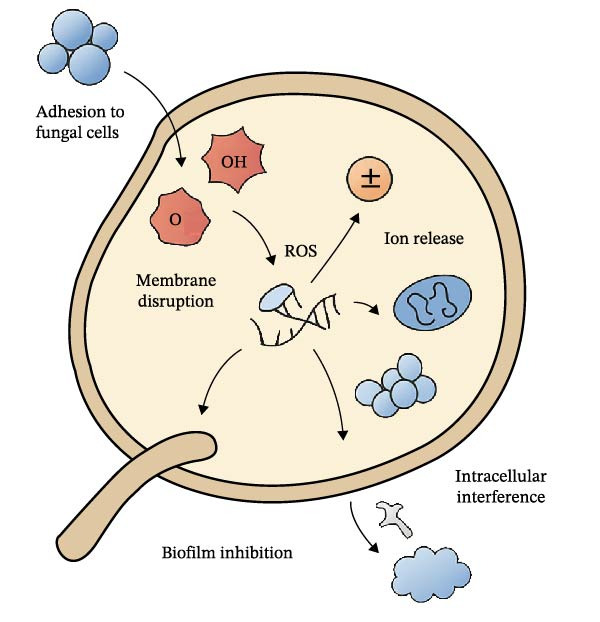
Schematic illustration of the multitarget mechanisms of antifungal NPs, including (1) adhesion to fungal cells, (2) membrane disruption, (3) ROS generation, (4) ion release, (5) intracellular interference, and (6) biofilm inhibition. This figure summarizes experimentally observed mechanisms reported across multiple antifungal NP studies.

One of the most prominent mechanisms is the disruption of fungal cell membrane and wall integrity. Many NPs, including silver, copper, zinc oxide, and chitosan, exhibit strong affinity for fungal surfaces due to electrostatic interactions between their positively charged surfaces and the negatively charged components of fungal membranes and cell walls, such as phospholipids, β‐glucans, and chitin. Once bound, NPs can insert into lipid bilayers, cause membrane thinning, induce leakage of ions and metabolites, and even form nanoscopic pores visible under electron microscopy [71]. Chitosan NPs, for example, bind to fungal glucans and chitin, impairing cell wall biosynthesis and collapsing hyphal membranes, a process particularly effective in filamentous fungi such as *Fusarium oxysporum*, *Botrytis cinerea*, *Penicillium expansum*, and *Aspergillus ochraceus* [72]. These interrelated actions are collectively illustrated in Figure 2, which depicts the major antifungal mechanisms of NPs at the fungal cell level.

### 3.2. Generation of ROS

Another conserved mechanism is the generation of ROS. Metal and metal oxide NPs, such as Ag, ZnO, CuO, and TiO_2_, can catalyze ROS formation either directly through surface redox reactions or indirectly by disrupting mitochondrial respiration. These ROS, including superoxide anions, hydroxyl radicals, singlet oxygen, and hydrogen peroxide, oxidize lipids, damage DNA, denature proteins, and interfere with nuclear and mitochondrial functions, often leading to apoptosis‐like cell death in fungi [73]. Experimental evidence shows that ROS scavengers such as glutathione can attenuate NP‐induced toxicity, underscoring oxidative stress as a primary mode of action [74].

### 3.3. Release of Toxic Metal Ions

In addition to ROS generation, inorganic NPs can release toxic metal ions such as Ag^+^, Cu^2+^, and Zn^2+^ into the surrounding environment. These ions bind to thiol groups in fungal enzymes, disrupt respiration and DNA synthesis, interfere with ATP generation, and displace essential cations such as Mg^2+^ and Fe^2+^, ultimately leading to metal stress and cell cycle arrest [75]. The rate of ion release depends on particle size, crystallinity, and pH, with smaller, more reactive NPs releasing ions more readily and thus exhibiting stronger antifungal effects. However, uncontrolled ion release can also increase phytotoxicity, making controlled‐release designs such as silica‐coated AgNPs preferable for agricultural use [76].

### 3.4. Intracellular Damage and Macromolecular Interference

Beyond surface‐level interactions, many NPs penetrate fungal cells through endocytosis or passive diffusion, accumulating within cytoplasmic or nuclear compartments. Once internalized, they can condense chromatin, fragment DNA, disrupt mitochondrial membranes, interfere with ribosomal protein synthesis, and modulate gene expression, particularly genes involved in oxidative stress response, apoptosis, and cell wall remodeling [77]. For example, AgNPs localize within the cytoplasm and nuclei of *Candida albicans*, inducing DNA condensation and nuclear fragmentation while suppressing hyphal development. Recent omics‐based studies further reveal that NPs can reprogram fungal transcriptomes and proteomes, including upregulation of oxidative stress genes, suppression of ergosterol biosynthesis pathways, and disruption of ribosomal protein translation, suggesting multilayered molecular responses that warrant deeper system‐level investigation.

### 3.5. Inhibition of Spore Germination and Hyphal Growth

Another important mechanism is inhibition of spore germination and hyphal growth. Spores represent the primary dissemination units of fungi, and NPs can adhere to spore surfaces, block hydration and metabolic activation, disrupt membranes, and induce morphological abnormalities in developing hyphae. Studies have shown that ZnO and CuO NPs can reduce the germination rates of *B. cinerea* and *F. oxysporum* spores by up to 90% in vitro, while field applications of Ag and ZnO NPs have suppressed lesion formation in wheat, tomato, and cucumber crops [78–80].

### 3.6. Inhibition of Biofilms and Quorum Sensing

Fungal biofilms present another major challenge in both clinical and agricultural settings due to their protective extracellular polymeric substances (EPSs) and metabolic heterogeneity. NPs counteract biofilms by penetrating the EPS matrix, disrupting quorum‐sensing signals, eradicating dormant fungal cells, and preventing adhesion on abiotic surfaces such as seeds, medical implants, or food packaging [81]. For instance, AgNPs have been shown to disrupt *Candida* biofilms by degrading β‐glucan–rich EPS and increasing azole susceptibility [82].

### 3.7. Synergy With Conventional Antifungals

**Figure 3:**
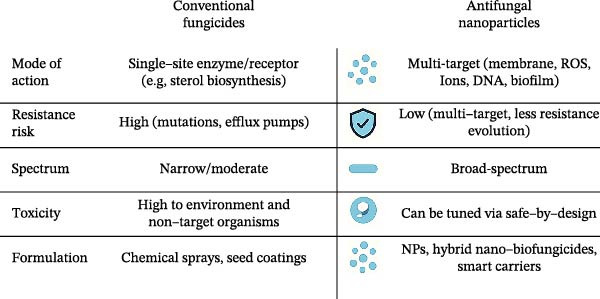
Comparison of antifungal NPs and conventional fungicides across key parameters: mechanism of action, resistance risk, environmental impact, and potential for synergistic combinations. NPs show multitarget activity and lower resistance likelihood compared to single‐site fungicides. This figure combines experimentally supported data with conceptual elements to provide an integrated overview.

Finally, NPs can act synergistically with conventional antifungals, restoring drug efficacy against resistant strains. Mechanisms of synergy include increasing membrane permeability to enhance drug uptake, inhibiting efflux pumps, and disrupting biofilms to improve penetration. For example, chitosan NPs have been shown to enhance fluconazole efficacy against *C. auris*, while AgNP‐amphotericin B hybrids exhibit combined ROS‐ and ion‐mediated killing [83–85]. Immobilization of biocontrol agents or enzymes on NP scaffolds has been shown to enhance stability and field efficacy. Representative NP‐microbe integrated systems with antifungal effects are summarized in Table 2. Such strategies lower the required drug dose, reducing systemic toxicity while improving therapeutic outcomes. A comparative overview of antifungal NPs and conventional fungicides in terms of spectrum, resistance development, and safety is illustrated in Figure 3.

**Table 2:** Nano‐biofungicide systems and their effects on crops.

NP + microbe	Target pathogen	Crop	Outcome	Key refs.
AgNPs + *Trichoderma*	*F. oxysporum*	Muskmelon	93% Suppression, 1.8 × biomass	[86, 87]
Chitosan + *Bacillus*	*B. cinerea*	Tomato	Reduced leaf lesions	[88, 89]
Alginate + *Pseudomonas*	Root rot fungi	Wheat	Enhanced root health	[90, 91]

In summary, antifungal NPs operate through diverse mechanisms, including cell membrane disruption, ROS generation, ion release, intracellular interference, spore and biofilm inhibition, and synergistic interactions with conventional antifungals. This multitarget activity not only increases their efficacy but also makes resistance evolution less likely, positioning NPs as key candidates for next‐generation antifungal strategies in agriculture, medicine, and microbial biotechnology. Table 3 presents a comparative overview of antifungal NPs tested against key pathogenic fungi.

**Table 3:** Comparative efficacy of antifungal NPs.

NP type	Fungal target	MIC (µg/mL)	Application mode	Key refs.
AgNPs (green tea)	*Candida albicans*	4.2	Topical/vaginal	[92]
ZnO NPs (aloe)	*Fusarium oxysporum*	15	Foliar spray	[93]
CuO NPs (Salvia)	*Botrytis cinerea*	25	Soil drench	[94]

## 4. Biomedical Applications (Human and Animal Health)

Although this section focuses on biomedical and clinical applications of antifungal NPs, these systems provide critical design principles that are directly transferable to agricultural nanobiofungicides and broader microbial biotechnology applications. Strategies extensively developed in biomedical contexts, such as NP‐mediated biofilm disruption, targeted and stimuli‐responsive delivery, synergistic antifungal‐carrier architectures, and resistance‐mitigation mechanisms, address challenges that are equally relevant in plant‐pathogen systems and agroecosystems. For instance, fungal biofilms on plant surfaces, seeds, and soil particles exhibit physicochemical and biological similarities to clinical biofilms, including extracellular polymeric matrices and adaptive resistance responses [95, 96]. Likewise, controlled‐release platforms, surface‐functionalized NPs, and combination therapies originally designed for clinical antifungal delivery can be rationally repurposed to improve field stability, dose efficiency, and selectivity of nanobiofungicides [97, 98]. Accordingly, the biomedical examples discussed in this section are presented not as a diversion from the agricultural focus but as translational models that inform next‐generation antifungal NP design across microbial biotechnology, agriculture, and food‐related systems.

Fungal infections in humans and animals present a growing clinical and public health challenge, particularly in immunocompromised patients, individuals in intensive care, and agricultural workers with frequent exposure to fungal spores. Opportunistic pathogens such as *C. albicans*, *C. auris*, *Aspergillus fumigatus*, *Cryptococcus neoformans*, and *Mucor* species are responsible for millions of life‐threatening infections annually, with mortality rates often exceeding 40% in invasive cases [99, 100]. Current antifungal therapies are limited by narrow spectra, poor pharmacokinetics, host toxicity, and increasing MDR, underscoring the urgent need for innovative strategies [101, 102]. The NPs, owing to their unique physicochemical properties, including high surface area‐to‐volume ratios, tunable surface functionalization, and capacity to cross biological barriers, offer new avenues for diagnosis, therapy, and drug delivery in mycology.

### 4.1. NPs as Therapeutic Agents for Resistant Fungal Infections

NPs themselves can act as therapeutic agents against resistant fungal infections. Metal‐based NPs such as silver, zinc oxide, and copper oxide disrupt fungal cells via ROS generation, ion release, and membrane damage, making them effective against MDR strains including *C. auris* [85]. For example, green‐synthesized AgNPs using plant extracts like green tea or neem have demonstrated minimum inhibitory concentrations below 5 µg/mL against azole‐resistant *C. albicans* and *C. auris* while also reducing fungal load and improving survival in murine systemic candidiasis models [103, 104]. Similarly, CuO NPs have shown potent antifungal activity against *Aspergillus* and *Mucor* species at concentrations nontoxic to mammalian cells, offering an affordable therapeutic option in resource‐limited settings [105]. These multitarget mechanisms lower the likelihood of resistance emergence, a major advantage over conventional drugs.

### 4.2. NP‐Enabled Drug Delivery Systems

Equally transformative are NP‐enabled drug delivery systems designed to overcome the limitations of existing antifungals. Lipid‐based formulations such as MAT2203, an oral lipid nanocrystal amphotericin B, encapsulate the drug in a lipid bilayer nanocrystal, enhancing oral bioavailability and reducing nephrotoxicity. MAT2203 has demonstrated efficacy in Phase 2 clinical trials for invasive mycoses, including mucormycosis and cryptococcosis, and has received FDA Fast Track designation [106]. Polymeric nanocarriers such as PLGA and chitosan NPs have been widely explored for controlled delivery of azoles and polyenes. For instance, PLGA‐based amphotericin B NPs reduced renal toxicity and improved CNS penetration in fungal meningitis models [107]. Chitosan‐based NPs, inherently antifungal and biocompatible, have been used to deliver essential oils and peptides for treating vulvovaginal candidiasis and dermatophytoses [108].

### 4.3. Combination Nanotherapies and Synergistic Systems

Synergistic systems that combine NPs with existing antifungals represent another promising frontier. NPs enhance drug efficacy by permeabilizing membranes, inhibiting efflux pumps, and improving intracellular accumulation. For example, albumin‐encapsulated minocycline NPs combined with fluconazole exhibited strong synergy against azole‐resistant *C. albicans*, significantly improving fungal clearance and survival in murine models compared to either agent alone [109]. Similarly, coloaded nanocarriers encapsulating two antifungals, such as amphotericin B and posaconazole, offer dual‐mechanism therapy with broader activity and reduced dosage requirements.

### 4.4. Topical and Mucosal Nanotherapies

Topical and mucosal nanotherapies are especially relevant for common but recurrent superficial fungal infections. NP sprays, gels, and films improve skin and mucosal penetration, reduce systemic side effects, and increase patient compliance. Clinical studies have already demonstrated their superiority; for instance, a randomized trial in Iran showed that a 15 ppm AgNP vaginal spray achieved a 98% clinical cure rate in vulvovaginal candidiasis compared with 67.9% for clotrimazole cream, with faster symptom relief and higher patient satisfaction [110]. Likewise, AgNP‐infused wound dressings are being developed to prevent fungal coinfections in diabetic ulcers and surgical wounds [111].

### 4.5. NPs in Veterinary and Environmental Mycoses

NPs also hold promise in veterinary and environmental mycoses. In livestock, chitosan‐AgNP composites have been successfully used to treat dermatomycoses such as ringworm in cattle, while ZnO NPs are being tested to control Saprolegnia infections in aquaculture [112]. In poultry and avian species, inhalable nanocarriers are being evaluated for respiratory aspergillosis. Beyond direct treatment, nanoenabled air filtration systems incorporating Ag or TiO_2_ NPs are being deployed in hospitals, food storage facilities, and agricultural environments to reduce airborne fungal spores such as *Aspergillus* and *Penicillium* [113, 114].

Taken together, biomedical applications of antifungal NPs now span prevention, treatment, and delivery across human, veterinary, and environmental contexts. Translational breakthroughs include the clinical development of MAT2203 as the first oral amphotericin B formulation, synergistic systems such as Min‐NP plus fluconazole, AgNP‐based sprays with proven clinical efficacy, and chitosan‐based carriers for mucosal therapy. In veterinary and aquaculture systems, nanoformulations are emerging as practical antifungal tools. These advances demonstrate that NP‐based antifungal systems have moved beyond conceptual frameworks and are entering clinical and agricultural practice. The challenge ahead lies in scaling these innovations, ensuring regulatory approval, and optimizing safety, especially in immunocompromised populations and ecologically sensitive settings.

## 5. Agricultural Applications of Antifungal NPs

Fungal phytopathogens remain among the most damaging threats to agricultural productivity, compromising yields, quality, and global food security. Pathogens such as *Fusarium* spp., *B. cinerea*, *A. solani*, *Colletotrichum gloeosporioides*, *Magnaporthe oryzae*, and *Verticillium dahliae* infect roots, stems, foliage, and fruits, causing wilting, rot, and blight that result in massive economic losses. Globally, fungi are estimated to account for 10%–23% of crop losses during the growing season and an additional 10%–20% postharvest, representing billions in food and economic waste each year [3]. The challenge is further compounded by the rapid emergence of fungicide resistance, largely driven by extensive use of single‐site inhibitors such as demethylation inhibitors (DMIs), quinone outside inhibitors (QoIs), and succinate dehydrogenase inhibitors (SDHIs) [115]. This resistance not only undermines long‐term chemical efficacy but also demands alternative or complementary antifungal strategies.

Nanotechnology offers such an alternative by providing multifunctional antifungal agents with tunable physicochemical properties. These systems reduce chemical load, extend persistence, and improve compatibility with microbial biocontrol. Antifungal NPs can function as direct fungicides, smart delivery systems for natural products or agrochemicals, synergistic coformulants with beneficial microbes, and postharvest preservatives that extend produce shelf life [25, 116].

### 5.1. Preharvest Crop Protection (Foliar, Soil, and Root Treatments)

In preharvest applications, NPs are deployed via foliar sprays, soil drenches, or seed coatings. AgNPs and ZnO NPs, particularly those synthesized using green routes such as *Aloe vera* or lemongrass extracts, have demonstrated strong suppression of *Botrytis*, *Fusarium*, and *Colletotrichum* [117]. Their antifungal activity is linked to inhibition of conidial germination and induction of oxidative stress in hyphae. In several cases, these treatments also promoted photosynthetic efficiency. Biogenic AgNPs reduced leaf lesion areas by up to 80% in tomato and cucumber greenhouse trials without visible phytotoxicity [118]. Similarly, copper oxide NPs derived from *Salvia officinalis* or microbial synthesis inhibited root‐zone colonization in tomatoes and cereals, while chitosan NPs provided dual benefits by both suppressing pathogens and inducing plant immunity through activation of pathogenesis‐related (PR) genes and chitinases [119, 120]. Advances in nanoemulsions, hydrogels, and polymer nanospheres are further improving foliar formulation stability, rainfastness, and controlled release [121].

### 5.2. Seed Treatments and Germination Protection

Seed treatments represent another crucial application, since seeds serve as major vectors of fungal pathogens. NP priming or nanocoating has been shown to reduce seed‐borne infections, improve germination, and protect seedlings from early soil pathogens. Mesoporous silica NPs loaded with natural antifungals such as eugenol suppressed *B. cinerea* in cucurbits while enhancing seedling vigor [90]. Chitosan–ZnO NP coatings reduced Fusarium incidence in wheat and maize by up to 60%, while hybrid silica–Ag NPs provided antifungal protection without impairing germination rates [122]. Green‐synthesized NPs, often produced via moringa or neem extracts, are especially attractive due to low toxicity and compatibility with beneficial microbes.

### 5.3. Nanobiofungicides: Integrating NPs With Microbial Biocontrol Agents

A major recent innovation is the integration of NPs with microbial biocontrol agents to form nanobiofungicides. These hybrid systems combine the broad‐spectrum efficacy of NPs with the ecological precision of antagonistic microbes such as *Trichoderma*, *Bacillus*, and *Pseudomonas*. For instance, AgNPs synthesized by *T. harzianum* combined with live *T. koningiopsis* spores suppressed *F. oxysporum* in muskmelon fields, increasing plant biomass by nearly twofold compared to untreated controls [86, 123, 124]. Similarly, *Bacillus*‐derived metabolites encapsulated in chitosan NPs showed extended shelf life and field stability relative to free metabolites [125]. Such hybrid formulations embody the goals of microbial biotechnology by enhancing pathogen suppression. At the same time, they help preserve soil microbial biodiversity. Such formulations highlight how microbial biotechnology can scale antifungal nanotech from lab to field by stabilizing beneficial microbes and reducing fungicide input.

### 5.4. Postharvest Applications: Packaging and Shelf‐Life Extension

In postharvest contexts, NP‐based edible coatings, sprays, and packaging films are being used to reduce fungal spoilage of perishable produce. Chitosan–ZnO films extended the shelf life of strawberries by up to 10 days at room temperature by suppressing *Rhizopus stolonifer* and *B. cinerea* [126–128]. AgNP‐embedded polyethylene and cellulose‐based films inhibited mold growth on bread and cheese, prolonging shelf stability by 40%–60% [129, 130]. Newer smart packaging systems are being designed with fungi‐responsive triggers that release antifungals in response to humidity or enzymatic activity, offering low‐cost, passive spoilage prevention in regions without reliable cold‐chain infrastructure [131].

### 5.5. Soil Compatibility and Microbiome Considerations

**Figure 4:**
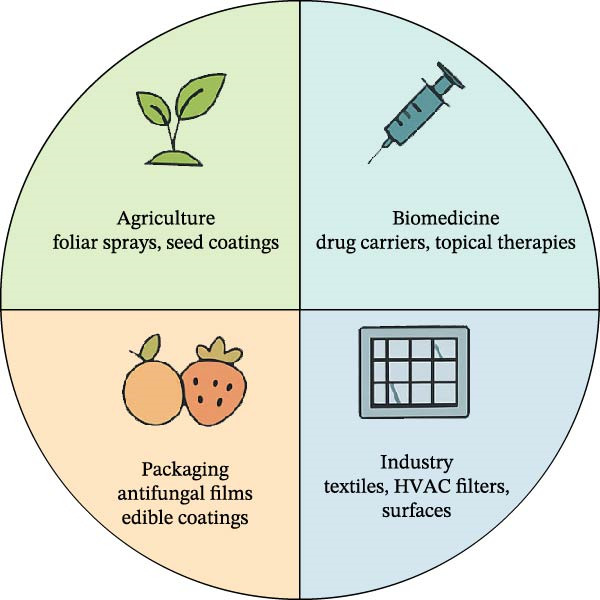
Sector‐specific applications of antifungal NPs, spanning agriculture, biomedicine, packaging, and industrial systems. Each domain utilizes NPs for unique goals, from disease suppression and seed protection to smart packaging, antifungal textiles, and therapeutic nanocarriers. This figure represents a conceptual application informed by recent published works.

Equally important are the ecological considerations of NP use. Soil represents a highly dynamic microbial ecosystem. Therefore, antifungal interventions must minimize disruption to beneficial microbiota. Green‐synthesized NPs such as sulfidized AgNPs or ZnO capped with plant metabolites have been shown to exert limited toxicity to nontarget organisms and, in some cases, enhanced enzymatic activity and nitrogen‐fixing bacterial abundance [132]. Biodegradable carriers like chitosan, cellulose, and alginate further reduce NP persistence, making them more compatible with sustainable or organic farming systems. Nonetheless, soil‐specific assessments remain essential, and SbD strategies, such as NPs that degrade after pathogen contact, are being developed to balance efficacy with ecological safety [133, 134]. Since microbial biotechnology increasingly emphasizes microbiome‐aware crop protection, future antifungal NP designs must balance pathogen suppression with preservation of beneficial taxa such as *mycorrhizae*, *Rhizobium*, and *Bacillus* species, ideally visualized as a soil ecosystem network where NPs selectively target pathogens while maintaining microbial diversity. The multifunctional use of antifungal NPs across diverse sectors is summarized in Figure 4, illustrating their deployment from field to clinic.

While many studies qualitatively describe microbiome modulation by antifungal NPs, emerging quantitative evidence from high‐throughput sequencing provides important benchmarks for microbiome‐aware nanobiofungicide design. These analyses indicate that low‐dose metallic (such as Fe and Zn) and polymeric NPs typically induce modest reductions in microbial α‐diversity, often within the range of ~5%–20% while selectively suppressing pathogenic fungal taxa [135]. Relative abundance shifts of dominant bacterial or fungal genera are commonly reported in the range of ±10%–30%, depending on NP composition, surface chemistry, and exposure duration [136]. Enzyme activity assays further suggest that soil or rhizosphere enzymatic functions (e.g., dehydrogenase and phosphatase) remain largely preserved or can even be stimulated at sublethal NP concentrations, with activity changes frequently below 15%–25% [137]. This is particularly evident for Fe‐ and Zn‐based NPs, which often show good biocompatibility with soil enzymes [138]. These findings collectively indicate the existence of a concentration‐dependent response window in which pathogen suppression can be achieved with limited disruption of beneficial microbial communities and their biochemical functions.

**Figure 5:**
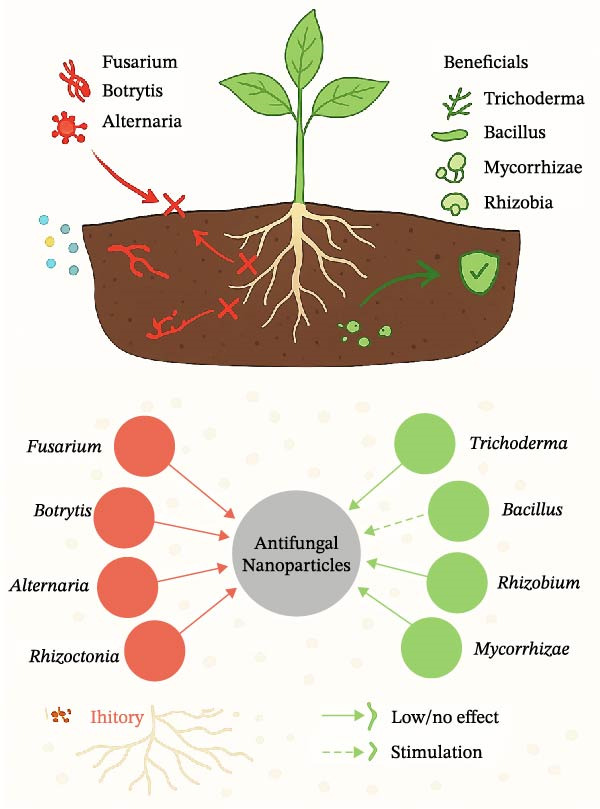
Schematic of NP‐microbiome interactions. While antifungal NPs suppress phytopathogens such as *Fusarium* and *Botrytis*, SbD approaches aim to preserve beneficial taxa, including *Trichoderma*, *Bacillus*, *mycorrhizae*, and *Rhizobia*, ensuring ecological balance. This figure represents a conceptual framework informed by reported experimental trends in microbiome‐NP interactions.

Figure 5 conceptually illustrates how antifungal NPs affect both pathogenic and beneficial microbes in soil ecosystems. Antifungal NPs are reshaping plant disease management across all stages of crop production, see Table 4. In summary, antifungal NPs are reshaping plant disease management across the entire agricultural cycle, from seed to storage. Their potential lies not only in reducing dependence on chemical fungicides but also in integrating with microbial biocontrol and smart packaging technologies. As regulatory frameworks mature and field trials confirm efficacy and safety, nanoenabled antifungal systems are poised to become key tools in precision agriculture. The next frontier will focus on intelligent nanosystems that can sense, respond, and degrade in situ, ensuring efficacy against pathogens while safeguarding ecological and food system resilience.

**Table 4:** Antifungal NPs are reshaping plant disease management across all stages of crop production.

Application	NP examples	Target pathogens	Benefits	Key refs.
Foliar sprays	Ag, CuO, ZnO, chitosan	*Fusarium*, *Botrytis*, *Alternaria*	Broad‐spectrum, low‐dose, persistent	[139, 140]
Seed coatings	Silica, chitosan–ZnO, AgNPs	*Fusarium*, *Botrytis*, *Verticillium*	Early‐stage protection, enhanced vigor	[141, 142]
Biofungicide hybrids	NPs + *Trichoderma/Bacillus*	Soil‐borne fungi	Synergistic, eco‐safe, microbiome‐friendly	[141, 143]
Postharvest packaging	ZnO, AgNPs, TiO_2_ composites	Storage molds	Shelf‐life extension, passive protection	[144, 145]
Soil health management	Green‐synthesized NPs	General soil pathogens	Nutrient enhancement, microbial balance	[25, 143]

## 6. Industrial and Packaging Applications of Antifungal NPs

Beyond their roles in clinical and agricultural systems, antifungal NPs are increasingly deployed in industrial, packaging, and environmental applications where fungal contamination compromises product integrity, safety, and shelf life. Fungal colonization on food‐contact surfaces, building materials, textiles, or ventilation systems can accelerate spoilage, weaken infrastructure, or pose health hazards. The incorporation of antifungal NPs into coatings, films, filters, and construction materials offers a proactive strategy to suppress fungal growth without continuous chemical treatments, aligning with sustainable material science and microbial biotechnology [146].

### 6.1. Food Packaging and Edible Coatings

Food spoilage caused by *Aspergillus niger*, *Penicillium* spp., and *R. stolonifer* remains a critical challenge across global supply chains. Mold growth not only leads to visible deterioration but also increases the risk of mycotoxin contamination, with implications for food safety and regulatory compliance. NPs are now incorporated into smart packaging films, edible coatings, and surface sprays to prolong shelf life and reduce the reliance on synthetic preservatives [22, 147]. ZnO NPs embedded in cellulose, chitosan, or polyethylene matrices inhibit fungal growth through ROS generation and Zn^2+^ ion release, while AgNPs provide strong fungicidal activity at low concentrations and are increasingly blended into biodegradable polymers such as PLA or starch‐based films [148, 149]. Hybrid films that combine AgNPs with ZnO or chitosan show synergistic antifungal performance while minimizing NP migration into food. Chitosan‐based edible coatings reinforced with ZnO or CuO NPs have delayed mold development on strawberries, blueberries, tomatoes, and citrus fruits by 7–14 days without refrigeration [128, 150]. Emerging “smart packaging” innovations incorporate fungal‐responsive triggers. For instance, curcumin‐loaded lipid NPs embedded in films released their active payload only under fungal‐secreted pectinase activity, thereby inhibiting *Aspergillus* growth without premature release [151]. Other systems integrate colorimetric sensors with ZnO or GO, enabling visual detection of fungal spoilage via pH‐sensitive dyes [152]. These multifunctional approaches highlight the shift toward active and intelligent packaging solutions designed to enhance food safety while reducing waste.

### 6.2. Antifungal Textiles, Paints, and Building Materials

High‐humidity environments promote fungal growth on building materials, textiles, and paints, leading to structural degradation and health hazards associated with indoor molds such as *Stachybotrys chartarum* and *Aspergillus flavus*. NP‐infused paints and coatings are being developed to provide persistent antifungal protection. For example, TiO_2_ and ZnO NPs incorporated into interior paints generate ROS under UV or visible light, preventing spore adhesion and hyphal proliferation [153]. AgNP‐enriched plasters are undergoing trials in hospitals and schools to reduce surface contamination in high‐contact areas. In textiles, AgNPs and chitosan–ZnO composites impart durable antifungal resistance to cotton, polyester, and silk fabrics, even after repeated washing. Such treatments are being applied in medical garments, sportswear, and protective clothing for agricultural workers [154]. Layer‐by‐layer assembly techniques allow NP coatings with enhanced wash durability, while eco‐friendly construction materials such as hempcrete and bamboo are increasingly treated with Ag‐ZnO hybrids to prevent fungal colonization without volatile biocides.

### 6.3. Air Purification and HVAC Systems

Airborne fungal spores are a significant concern in indoor environments, particularly within HVAC systems, cold storage facilities, and greenhouses. Exposure can cause allergic disease, asthma, and occupational respiratory syndromes. NP‐coated air filters are being developed as passive antifungal barriers. AgNP‐ and TiO_2_‐coated filters installed in HVAC ducts effectively suppressed *Aspergillus*, *Penicillium*, and *Rhizopus* spores while maintaining airflow [155]. Photocatalytic TiO_2_ systems generate continuous ROS under UV‐C illumination, offering long‐term decontamination. GO coatings add mechanical reinforcement and supporting antimicrobial effects, improving filter longevity [156]. These NP‐coated systems provide maintenance‐free protection, reducing reliance on ozone sterilization or chemical fumigation while safeguarding air quality in sensitive environments such as hospitals and food‐processing units.

### 6.4. Antifungal Surfaces for Industrial and Laboratory Settings

Industrial and laboratory surfaces must resist fungal colonization to prevent contamination of pharmaceuticals, food products, and biotechnological cultures. Stainless steel, polypropylene, and polymer surfaces coated with AgNPs or ZnO‐silica composites have demonstrated strong antifungal activity and antibiofilm properties, even under abrasive and sterilization conditions [157]. In cleanroom settings, antifungal tiles and benches infused with PLGA‐AgNP composites are being tested for long‐term durability. Additive manufacturing enables 3D‐printed antifungal surfaces with embedded NPs, allowing customized designs for vertical farming systems, bioreactors, and specialized laboratory equipment. These innovations provide adaptable solutions for niche industrial needs.

### 6.5. Water Treatment and Agricultural Storage Systems

Fungal contamination in water storage tanks, irrigation systems, and hydroponic setups reduces water quality and introduces pathogens into crops. NP‐based antifungal systems offer preventive solutions in these environments. Ceramic and polymer filters infused with Ag, CuO, or ZnO NPs inhibit fungal proliferation, while antifouling nanocoatings on irrigation pipes prevent biofilm formation [158]. Nanostructured membranes are also being integrated into portable water purification systems for farms in humid regions where fungal growth is accelerated. Industrial and packaging applications of antifungal NPs highlight their versatility across food preservation, building materials, textiles, HVAC systems, and water treatment. Table 5 summarizes key NP‐enabled systems. The major advantages of NP‐enabled systems include long‐lasting durability, passive antifungal protection, and compatibility with sustainable materials. However, widespread adoption requires addressing regulatory concerns regarding NP migration, environmental persistence, and long‐term safety [28, 165]. Future directions emphasize smart nanomaterials that respond selectively to fungal metabolites, multifunctional coatings with combined antifungal, antibacterial, and antiviral properties, and biodegradable formulations suitable for circular economy models. With standardized safety frameworks, antifungal nanotechnologies are positioned to redefine fungal control in industrial, commercial, and consumer environments.

**Table 5:** Summarizes key NP‐enabled systems.

Application area	NPs used	Target fungi	Key benefits	Key refs.
Food packaging	ZnO, Ag, chitosan	*Penicillium*, *Aspergillus*, *Rhizopus*	Mold prevention, shelf‐life extension	[159, 160]
Edible coatings	ZnO‐chitosan, lipid NPs	*Botrytis*, *Fusarium*	Safe, GRAS‐approved, biodegradable	[161]
Antifungal paints	TiO_2_, ZnO, Ag	Indoor molds	Photocatalytic protection, long‐lasting	[24, 158]
Antimicrobial textiles	AgNPs, chitosan–ZnO	*Aspergillus*, *Trichophyton*	Washable, breathable, antifungal clothing	[158, 162]
Air purification systems	TiO_2_, AgNPs, GO	Airborne spores	Passive, low‐energy sterilization	[163]
Water systems	CuO, AgNPs, ZnO	*Rhizopus*, *Cladosporium*, *Fusarium*	Antifungal filtration, biofilm prevention	[145, 164]

## 7. Safety, Toxicity, and Environmental Implications of Antifungal NPs

As antifungal NPs progress toward clinical, agricultural, and industrial deployment, their safety and ecological impact have become central concerns. Their nanoscale dimensions, high surface reactivity, and potential persistence in the environment provide unparalleled antimicrobial potency but also raise risks of unintended interactions with nontarget organisms, human tissues, and ecological systems [166]. Risk assessment must therefore address acute and chronic toxicity, environmental accumulation, and NP transformation [167, 168]. Particular attention is required for impacts on microbial communities in soil, where microbial balance underpins plant health and ecosystem function.

### 7.1. Determinants of NP Toxicity

The toxicity of antifungal NPs depends strongly on their physicochemical properties. Core composition (Ag, CuO, ZnO, TiO_2_, and chitosan), size, shape, and surface charge dictate bioactivity, with smaller and positively charged particles often showing higher membrane‐disruptive capacity [169, 170]. Dissolution potential and ion release (e.g., Ag^+^ and Cu^2+^) are critical mediators of cytotoxicity, while agglomeration and the formation of a biomolecular corona (proteins, polysaccharides, and humic acids) influence uptake and bioavailability [171]. Comparative studies generally rank AgNPs as the most cytotoxic, followed by CuO and ZnO, whereas TiO_2_ and biopolymer‐based NPs (e.g., chitosan and alginate) are relatively biocompatible and biodegradable [172, 173]. Toxic thresholds vary by system; for example, AgNPs above 50 ppm induce apoptosis in mammalian cells [174], ZnO NPs are tolerated in plants below ~ 20 ppm [175], and CuO NPs trigger oxidative stress in aquatic organisms at 10–30 ppm [176].

### 7.2. Human and Animal Health Concerns

In medical and veterinary applications, toxicity manifests as organ stress, inflammation, or bioaccumulation. In vitro assays show that AgNPs impair mitochondrial function and DNA repair in fibroblasts and hepatocytes, ZnO NPs remain largely safe below 10 µg/mL [177, 178] but cytotoxic above 30 µg/mL, and CuO NPs induce hemolysis and hepatotoxicity in rodents [179]. By contrast, chitosan NPs demonstrate favorable safety profiles and are already used in FDA‐approved wound dressings and hemostats. The in vivo, toxicity depends on dose, administration route, coating, and clearance. PEGylated or polymer‐coated AgNPs are more readily cleared from organs, while oral lipid nanocrystal formulations of amphotericin B (MAT2203) have significantly reduced nephrotoxicity compared to intravenous delivery [180, 181]. Regulatory agencies such as the European Medicines Agency (EMA) and FDA require comprehensive evaluation of pharmacokinetics, genotoxicity, and reproductive safety, though no antifungal NP–based drugs have yet received full approval despite several entering Phase II trials.

### 7.3. Plant and Crop Toxicity

In agriculture, NP toxicity to crops and plant‐associated microbiota is a double‐edged issue: at optimized doses, NPs can improve plant growth and immunity, but excessive exposure may induce phytotoxicity [182]. For example, ZnO NPs enhance chlorophyll content, seed germination, and yield in cereals [183], while chitosan NPs induce systemic resistance pathways in tomato and grapevine [184]. Conversely, high levels of CuO NPs (>100 mg/kg soil) reduce rice seedling elongation [185], ZnO NPs at elevated concentrations cause oxidative stress in lettuce [186], and AgNPs above 200 mg/kg inhibit nutrient uptake in legumes [187]. Application method and formulation (seed coating, foliar spray, or soil drench) are therefore critical variables in balancing efficacy with safety.

### 7.4. Soil Microbiome and Ecosystem Effects

The soil microbiome is central to nutrient cycling, plant‐microbe symbiosis, and pathogen suppression. Evidence from recent studies indicates that low doses of biogenic or sulfidized NPs can minimize disruption or even stimulate beneficial taxa [188]. For instance, microbial‐synthesized AgNPs promoted Actinobacteria and Proteobacteria in the rhizosphere [189, 190], while green ZnO NPs exhibited minimal interference with nitrogen‐fixing bacteria [191]. Sulfidized AgNPs release fewer ions, reducing toxicity [192], whereas uncoated CuO NPs suppressed enzymatic activities such as urease and phosphatase, compromising soil function [193]. Overall, nanofungicides typically require lower metal inputs than bulk fungicides, potentially reducing environmental load, but repeated or high‐dose applications risk cumulative effects on microbial communities. Longitudinal metagenomic and metabolomic monitoring of NP‐treated soils could provide critical insight into resilience and recovery of microbial communities, bridging nanotoxicology with microbial ecology in ways highly relevant for microbial biotechnology.

From an ecological safety perspective, multiple studies suggest the presence of operational dose windows for antifungal NPs, in which effective pathogen inhibition occurs without irreversible microbiome perturbation [194]. At low to moderate NP loadings, antifungal efficacy is often achieved through localized interactions with fungal membranes or biofilms, while broader microbial networks remain functionally resilient. This aligns with the observation that certain nanomaterials, like Fe and Zn citrate NPs, can stimulate microbial populations and enzyme activities at agronomically relevant doses [138]. In contrast, higher concentrations or repeated exposure cycles may lead to nonselective antimicrobial effects, reduced microbial diversity, and altered nutrient cycling dynamics [137]. These observations highlight the importance of dose optimization, exposure frequency control, and NP degradability as central design criteria for microbiome‐compatible nanobiofungicides.

### 7.5. Aquatic and Pollinator Safety

Aquatic systems are particularly sensitive to NP leaching. AgNPs and ZnO NPs can inhibit algal photosynthesis at concentrations >100 µg/L [195], while CuO NPs induce oxidative stress and liver damage in zebrafish at 50–100 µg/L [196]. Encapsulation and sulfidation strategies mitigate these risks by lowering ion release and bioavailability. Pollinator safety remains underexplored, though early studies suggest that AgNPs may disrupt bee gut microbiota at high doses [197], while chitosan‐based nanocarriers are nontoxic and may even support bee health when incorporated into supplemental feed [198]. Expanding ecotoxicological studies to pollinators and other keystone species is essential before widescale agricultural deployment.

### 7.6. Environmental Fate and Transformation

Once released into the environment, NPs undergo transformations that determine their persistence and ecological impact. Soil pH, ionic strength, and organic matter influence aggregation and dissolution. Humic acids reduce metal ion release by binding NPs [199], while photochemical degradation affects polymer‐based carriers [200]. Chitosan and alginate matrices typically degrade within 7–30 days in soil [201], whereas PEGylated or silica‐coated particles persist longer but remain less mobile [202]. AgNPs frequently transform to Ag_2_S in soils, a relatively inert phase with low bioavailability [203]. Metal ions may adsorb to clays or organic matter, limiting leaching but potentially creating long‐term reservoirs of stress if overapplied. Life cycle assessment (LCA) models are increasingly used to predict cumulative environmental impacts of nanoenabled fungicides.

### 7.7. SbD Approaches

**Figure 6:**
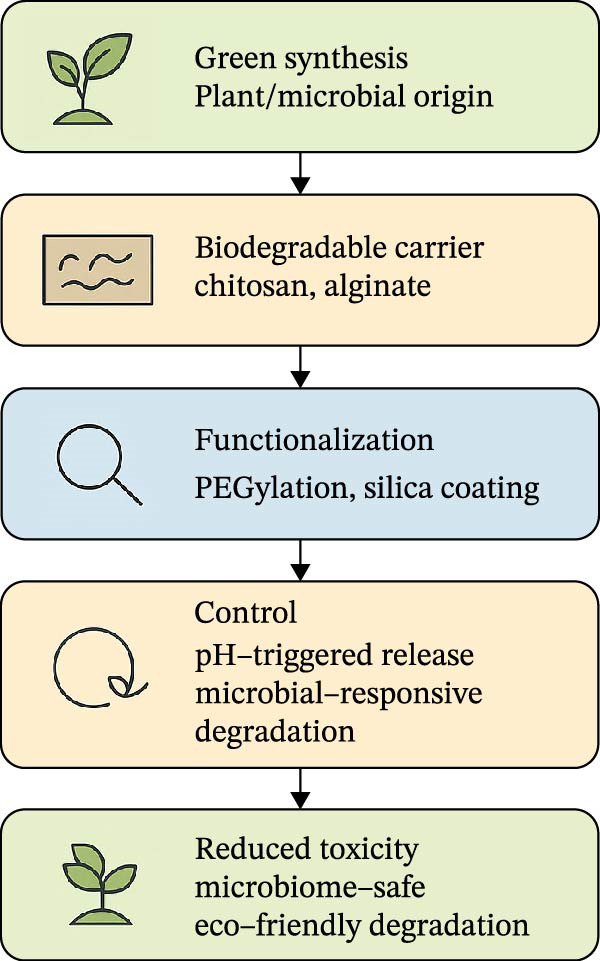
The SbD strategies for antifungal NPs include green synthesis using plant/microbial extracts, biodegradable carriers (chitosan and alginate), ion‐release control (silica coating and sulfidation), surface functionalization, and integration with beneficial microbes to mitigate toxicity. Informed by experimental data, this figure illustrates a conceptual framework for understanding key trends in microbiome‐NP interactions.

To reconcile efficacy with safety, researchers are adopting SbD strategies. These include green synthesis routes using plant or microbial extracts, biodegradable carriers such as chitosan or alginate, and surface modifications (PEGylation, silica shells, and sulfidation) to control ion release. Adjusting size and charge reduces nontarget interactions, while prescreening toxicity through computational modeling (e.g., QSAR and machine learning (ML)) can streamline safety assessment [204–206]. Integration with beneficial microbes such as *Trichoderma* or *Bacillus* reduces chemical input while enhancing ecological compatibility [207]. Regulatory‐aligned testing under OECD guidelines is essential for soil, water, and nontarget organisms, ensuring robust safety data prior to approval. To minimize environmental and biological risks, multiple SbD strategies for antifungal NPs are depicted in Figure 6. Antifungal NPs hold transformative promise but carry inherent risks that must be addressed proactively. Their toxicity varies by composition, coating, and dose, with AgNPs and copper NPs generally more hazardous than zinc oxide or biopolymer‐based systems. Environmental safety can be enhanced through green synthesis, biodegradable carriers, and controlled‐release formulations. However, further research is still needed on pollinators and aquatic ecosystems. Moving forward, the next generation of antifungal nanomaterials must embody precision, maximizing pathogen control while minimizing collateral damage to human health and ecosystems. Regulatory frameworks are evolving, but embedding SbD principles and LCA into early development will be essential to ensure responsible scaling and societal trust.

## 8. Regulatory Frameworks and Translational Considerations

Despite the demonstrated efficacy and broad‐spectrum potential of antifungal NPs, their translation from laboratory innovation to real‐world deployment remains constrained by regulatory complexity. Unlike conventional fungicides or pharmaceuticals, nanomaterials often occupy a regulatory “gray zone,” as their size‐dependent properties and dynamic behaviors complicate risk assessment and long‐term safety predictions. Consequently, regulators face the challenge of adapting existing frameworks, originally designed for bulk chemicals or biologics, to evaluate nanoenabled products.

### 8.1. Overview of Existing Regulatory Frameworks for Antifungal NPs

Despite the demonstrated efficacy and broad‐spectrum potential of antifungal NPs, their translation from laboratory innovation to real‐world deployment remains constrained by regulatory complexity. Unlike conventional fungicides or pharmaceuticals, nanoenabled products often occupy a regulatory “gray zone,” as size‐dependent properties, surface reactivity, and dynamic environmental behavior complicate hazard identification and long‐term safety assessment. Consequently, existing regulatory frameworks (originally developed for bulk chemicals or biologics) are frequently adapted rather than specifically designed to evaluate antifungal nanomaterials. Regulatory classification of antifungal NPs is largely application‐driven rather than composition‐driven, meaning that identical NP formulations may fall under different regulatory pathways depending on their intended use. For example, AgNPs incorporated into seed coatings are regulated as pesticides, while similar particles used in edible fruit coatings are assessed under food safety legislation, and those employed in wound dressings fall under medical product frameworks [208]. This fragmented classification approach presents challenges for multifunctional nanoformulations that span agricultural, biomedical, and industrial sectors.

In the biomedical domain, antifungal nanomedicines in the United States are regulated by the FDA’s Center for Drug Evaluation and Research (CDER), which requires conventional efficacy and safety data alongside nanospecific parameters such as particle size distribution, surface charge, aggregation behavior, and biodistribution [209]. The EMA applies similar expectations for nanocarrier characterization. Importantly, several nanoenabled antifungal formulations (such as MAT2203, an oral lipid nanocrystal formulation of amphotericin B) have advanced into Phase II clinical trials, demonstrating the feasibility of existing regulatory pathways for well‐characterized systems. In agricultural applications, antifungal NPs are regulated as pesticides under the U.S. Environmental Protection Agency’s Federal Insecticide, Fungicide, and Rodenticide Act (FIFRA), where nanoscale formulations are typically treated as new active ingredients, even when their bulk counterparts are already approved. In the European Union, Regulation (EC) No. 1107/2009 governs plant protection products, complemented by EFSA’s 2021 nanotechnology guidance, which mandates extensive physicochemical, toxicological, and ecotoxicological evaluation of nanomaterials. Other major agricultural economies, including China, India, and Brazil, are developing nanopesticide regulatory frameworks, often aligned with OECD recommendations, though harmonization and implementation timelines remain inconsistent. For food packaging and edible coatings, NPs incorporated into food contact materials must meet strict migration and stability requirements. In the United States, these products are regulated under FDA Title 21 CFR 177, while in the European Union Regulation (EU) No. 10/2011 includes explicit nanospecific provisions. Commercial examples include AgNP‐ and ZnO‐based polymer films that comply with these regulations in select jurisdictions [210]. Table 6 summarizes sector‐specific regulatory frameworks relevant to antifungal NP products.

**Table 6:** Nano‐biofungicide systems requirements and their benefits.

Sector	Regulatory body	Main regulation	NP‐specific requirement	Example product	Key refs.
Agriculture	US EPA	FIFRA	Treated as new active ingredient	AgNP seed coat	[211]
Biomedicine	US FDA	CDER NDA pathway	Size distribution, biodistribution	MAT2203	[180]
Packaging	EFSA	EU No. 10/2011	Migration testing	AgNP‐PE film	[212, 213]

Across sectors, successful translation of antifungal NPs depends on meeting several shared regulatory checkpoints. These include standardized physicochemical characterization (such as particle size distribution, polydispersity, surface chemistry, ion release profiles, and shelf stability), preferably generated using recognized protocols such as OECD Test Guideline 125 and complementary techniques (DLS, TEM, SEM, and SAXS). Manufacturing scale‐up must demonstrate batch‐to‐batch consistency and regulatory compliance, which remains challenging for green synthesis routes that often lack reproducibility. Comprehensive toxicological profiling, including nanospecific endpoints across mammalian, soil, and aquatic systems, is increasingly expected, alongside environmental risk assessment addressing persistence, transformation, and microbial ecosystem impacts [25]. Finally, regulators and stakeholders increasingly consider cost–benefit justification and public perception; labeling practices indicating the presence of nanomaterials have been shown to influence consumer acceptance, underscoring the need for transparent communication and responsible risk disclosure [28].

### 8.2. Unresolved Regulatory Challenges for Nanobiofungicides

Despite well‑established frameworks for pesticides and antimicrobial agents, nanobiofungicides present unique challenges not fully addressed by current regulations [211]. A primary gap is the absence of universally accepted nanospecific definitions and classification criteria. This often leads to NP‑based biofungicides being assessed as conventional chemical pesticides or treated articles, without sufficient consideration of their size‑dependent behavior, surface reactivity, or environmental transformation pathways [214]. Existing risk‑assessment protocols predominantly emphasize acute toxicity and environmental persistence while largely neglecting microbiome‑level effects. Current European guidelines, for example, may require only a nitrogen‑mineralization test for soil microorganisms, overlooking broader impacts on nontarget soil, rhizosphere, and phyllosphere communities [215]. This oversight is significant because nanobiofungicides can alter microbial structure and function, as shown by high‑throughput sequencing studies that reveal shifts in α‑diversity and functional pathways [216].

Another unresolved challenge concerns dose metrics and exposure scenarios. Traditional mass–based thresholds may not adequately capture NP activity, where antifungal efficacy and ecological impact are governed by surface area, particle number, or dissolution kinetics. This mismatch complicates hazard evaluation and the establishment of safe application windows. Regulatory frameworks also typically evaluate multicomponent nanobiofungicides (such as active NP cores, polymeric carriers, and biological stabilizers) in isolation rather than as integrated systems, missing potential synergistic effects. Finally, regulatory pathways rarely account for the dynamic behavior of nanobiofungicides in complex agroecosystems, where NPs may undergo aggregation, dissolution, or biological transformation [217]. The lack of standardized test protocols for nanoenabled biofungicides (especially those designed to be biodegradable or microbiome‑compatible) represents a significant barrier to translation. Addressing these gaps will require adaptive regulatory approaches that integrate nanospecific characterization, microbiome‑aware risk assessment, and lifecycle‑based evaluation strategies.

### 8.3. Toward Regulatory Harmonization and Nanospecific Policies

Efforts in response to the challenges posed by nanomaterials, international regulatory harmonization, and nanotailored guidelines have gained traction. The OECD’s Working Party on Manufactured Nanomaterials has released test guidelines and guidance for characterizing engineered NPs, laying the foundation for internationally consistent safety assessments. In parallel, standardization efforts via ISO/TC 229 are advancing terminology, metrology, and safety practices specific to nanotechnologies, including nanopesticides and nanobiocides. The FAO/WHO is also working to standardize approaches for nanoenabled food and agricultural technologies, particularly for global harmonization and application in low‐ and middle‐income countries. Crucially, the regulatory paradigm is shifting toward an emphasis on SbD: Embedding safety, biodegradability, and ecological compatibility into nanomaterial design from the outset is increasingly seen as a prerequisite for streamlined approval and broader societal acceptance [218].

### 8.4. Lessons From Near‐Commercial Products

Although relatively few antifungal NP products are currently fully commercialized, several near‐market prototypes provide valuable lessons for successful translation. The oral lipid nanocrystal formulation of amphotericin B (MAT2203), for instance, has progressed into clinical trials by coupling reduced systemic toxicity with meticulous NP characterization [209]. A AgNP‐based vaginal spray has achieved market authorization in multiple countries after outperforming standard treatments in clinical comparisons [110]. Meanwhile, ZnO‐ and AgNP‐infused packaging films have entered select markets under compliance with migration and safety standards [170, 172]. Additionally, chitosan–ZnO coatings developed for postharvest applications in tropical export chains (e.g., bananas and mangoes) have passed both field performance and regulatory scrutiny [219–221]. These cases collectively highlight that early integration of robust characterization, real‐world durability data, environmental safety assessments, and field validation enhances the likelihood of regulatory success and real‐world impact.

The regulatory trajectory of antifungal NPs is just as critical as their scientific advancement, since translation into real‐world use requires more than efficacy data alone. Successful deployment depends on aligning product classification precisely with its intended use, adopting standardized characterization and testing protocols tailored to nanoscale materials, and demonstrating safety across both biological and environmental systems. Proactive engagement with regulatory agencies at early stages of development helps innovators navigate evolving approval pathways, while the adoption of SbD principles enhances sustainability and fosters public trust (OECD, 2023; ISO/TC 229, 2023). As global regulatory frameworks mature and harmonize, antifungal NPs that integrate performance with safety are well positioned to move from experimental promise to regulated practice, redefining fungal control in clinics, farms, food systems, and industrial supply chains.

## 9. Future Directions and Research Gaps

The past decade has seen remarkable advances in the development of antifungal NPs, with applications spanning agriculture, medicine, packaging, and environmental protection [222]. Yet, despite encouraging laboratory and pilot‐scale results, relatively few NP‐based antifungal products have achieved regulatory approval or widespread adoption. This translational gap reflects persistent challenges in mechanistic understanding, biosafety assessment, scalable production, and long‐term performance validation. To unlock the full potential of antifungal nanotechnology, especially within microbial biotechnology, a coordinated research agenda is needed.

### 9.1. Mechanistic Understanding at the Nanofungus Interface

Although antifungal NPs are known to act through membrane disruption, ROS generation, and ion release, the precise molecular responses of fungal cells remain poorly characterized. Future research should prioritize transcriptomic and proteomic profiling of fungi exposed to NPs at different doses and time scales, alongside studies dissecting NP‐triggered apoptosis, necrosis, and autophagy pathways in pathogenic and nonpathogenic fungi. Clarifying NP‐biofilm interactions, including penetration kinetics, EPS modulation, and quorum‐sensing disruption, is equally critical [223]. Comparative studies on the likelihood of fungal resistance development against NPs versus conventional fungicides would provide essential insight into whether nanomaterials truly offer resilience against resistance evolution. Such mechanistic clarity will enable rational NP design and guide synergistic use with existing antifungals. Incorporating fungal omics, advanced imaging, and computational modeling into NP–fungus interaction studies will not only clarify molecular mechanisms but also enable predictive design of nanomaterials with tailored antifungal activity and minimal ecological disruption.

### 9.2. Nanomicrobiome Interactions and Ecological Balance

As NP applications extend into soil, crop, and water systems, understanding their effects on nontarget microbial communities is critical. Beneficial microbes such as *Rhizobia*, *Trichoderma*, *Bacillus*, *mycorrhizae*, and nitrogen fixers underpin plant health and ecological resilience. Key priorities include longitudinal metagenomic studies of soil and rhizosphere communities under NP exposure, determination of thresholds that suppress pathogens without harming beneficial taxa, and assessment of microbial community resilience, horizontal gene transfer, and postexposure recovery. Parallel efforts should focus on developing NP‐microbial synergies that enhance beneficial taxa while suppressing fungal pathogens. These insights will support the design of microbiome‐compatible nanomaterials aligned with ecological sustainability. Visualizing these dynamics in schematic models, contrasting NP effects on pathogens versus beneficial microbes, would provide a powerful framework for designing microbiome‐compatible nanobiofungicides.

### 9.3. SbD Engineering and Biodegradability

Current antifungal NPs often rely on persistent inorganic cores such as Ag, Cu, or ZnO, raising concerns about bioaccumulation and long‐term ecotoxicity. Future development should emphasize biodegradable nanocarriers such as protein nanospheres, cellulose nanofibers, or DNA origami, together with stimuli‐responsive NPs that degrade upon exposure to fungal enzymes, pH shifts, or environmental triggers. Advanced surface modifications such as sulfidation or biopolymer capping can help control ion release and reduce off‐target toxicity. Meanwhile, computational modeling and ML approaches are beginning to offer predictive frameworks for designing optimized low‐toxicity nanostructures. These approaches align with regulatory expectations and will improve environmental and consumer safety.

### 9.4. Field Validation and Long‐Term Monitoring

Most current NP research remains confined to in vitro assays and short‐term greenhouse studies. Large‐scale, long‐term validation is essential to build regulatory confidence and farmer adoption. Multiseason, multisite field trials across diverse agroclimatic zones are required, along with monitoring of crop yield, disease incidence, NP residue levels, and soil microbiome health. Parallel innovation is needed in portable NP biosensors for real‐time tracking of nanomaterials in soil and water systems. Characterizing NP persistence and transformation under sunlight, rainfall, and microbial degradation will provide a realistic evidence base for cost–benefit analysis, agronomic optimization, and responsible stewardship.

### 9.5. Regulatory Innovation and Harmonization

As outlined in Section 8, regulatory frameworks for nanomaterials remain fragmented and inconsistent across jurisdictions. Harmonization and innovation are urgently needed, including cross‐national adoption of standardized nanospecific test protocols under ISO, OECD, EFSA, and FDA guidelines, recognition of SbD certification as a pathway for expedited approval, creation of open‐access data platforms for NP toxicity and environmental fate, and tailored guidance for hybrid nanobiocontrol products that integrate living microbes with nanoscale components. Clearer, harmonized policies will reduce development barriers and accelerate safe global deployment.

### 9.6. Integration With Synthetic Biology and Microbial Engineering

**Figure 7:**
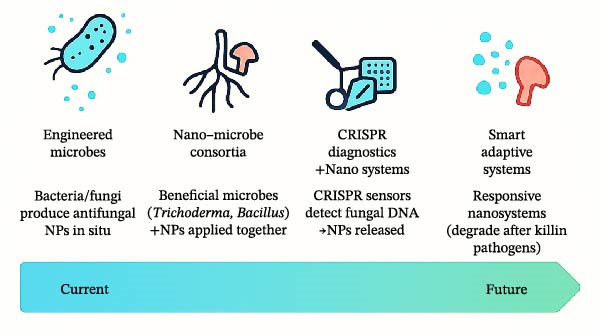
Roadmap for convergence of antifungal nanotechnology with microbial engineering and synthetic biology. Potential innovations include engineered microbes producing antifungal NPs in situ, nanomicrobe consortia for crop protection, CRISPR‐based fungal detection, and smart nanosystems for precision agriculture. This schematic is a conceptual framework synthesized from reported literature trends.

One of the most exciting frontiers lies at the convergence of nanotechnology with synthetic biology and microbial engineering. Synthetic biology platforms, such as engineered microbial scaffolds for pathway compartmentalization [224], highlight the potential of integrating microbial biotechnology with nanomaterials for adaptive antifungal systems. Microbial biotechnology may enable next‐generation antifungal systems that are adaptive, sustainable, and self‐regulating. Concepts now under exploration include engineered microbes capable of in situ biosynthesis of antifungal NPs at infection sites, rhizosphere bacteria delivering NP‐bioactive conjugates directly to plants, synthetic microbial consortia programmed to sense fungal pathogens and trigger NP release, and CRISPR‐based fungal sensors integrated with nanodiagnostic platforms. These hybrid approaches could redefine fungal disease management across agriculture, food systems, and healthcare. Figure 7 presents a forward‐looking roadmap where nanotechnology converges with synthetic biology to develop adaptive antifungal systems.

**Figure 8:**
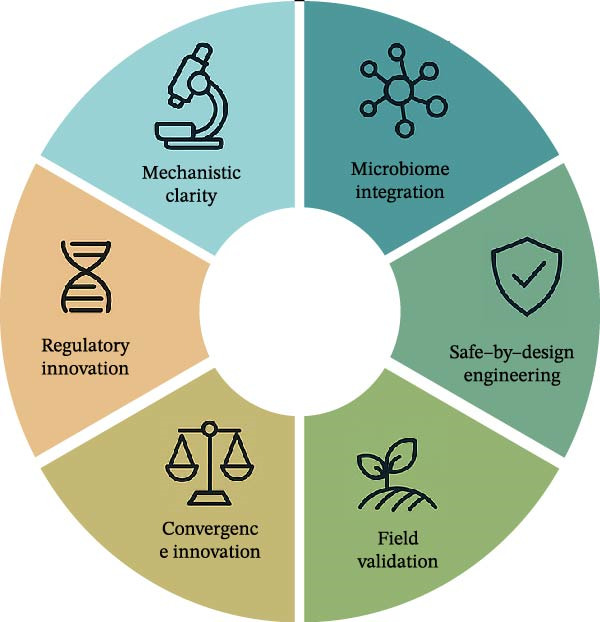
A future roadmap for antifungal nanotechnology detailing six strategic axes: (1) mechanistic clarity, (2) microbiome integration, (3) SbD engineering, (4) field validation, (5) regulatory innovation, and (6) convergence with synthetic biology and microbial engineering. This schematic presents a conceptual framework for future literature.

The future of antifungal nanotechnology depends on bridging critical knowledge and implementation gaps. Priority areas include mechanistic studies at the fungal and microbiome interface; eco‐compatible design using biodegradable and responsive nanomaterials; rigorous field validation across crops, climates, and time scales; regulatory harmonization supported by SbD pathways; and integration with microbial engineering, artificial intelligence, and synthetic biology. By aligning material science with microbial ecology and biotechnology, antifungal NPs can evolve from experimental tools into scalable, precision platforms in the global fight against fungal diseases. To synthesize these priorities, Table 7 summarizes a future roadmap for antifungal nanotechnology framed within microbial biotechnology, highlighting strategic axes, microbial contributions, and research needs. The envisioned roadmap for antifungal nanotechnology is illustrated in Figure 8, outlining critical research priorities and translational strategies.

**Table 7:** Future roadmap for antifungal nanotechnology within microbial biotechnology.

Strategic axis	Microbial biotech contribution	Key research Needs
Mechanistic clarity	Microbial omics reveal NP‐fungus interactions	Transcriptomics/proteomics of NP‐treated fungi
Microbiome integration	Soil/rhizosphere monitoring, beneficial microbes	Long‐term metagenomic studies, microbiome‐aware NP design
SbD engineering	Microbial/green NP synthesis, biodegradable carriers	Biopolymer‐based NPs, enzyme‐triggered degradation
Field validation	Microbial consortia + NP field trials	Multiseason, multisite validation
Regulatory innovation	Microbial‐NP hybrids as new regulatory category	Harmonized OECD/ISO nanobiopesticide guidelines
Convergence with synthetic biology	Engineered microbes as NP factories, NP‐microbe consortia	CRISPR‐based sensing + in situ NP biosynthesis

*Note*: The table highlights six strategic axes, the microbial biotechnology contribution for each, and corresponding key research needs.

## 10. Conclusions

Antifungal NPs represent more than a new class of fungicides, and they are an enabling technology for microbial biotechnology. By leveraging biogenic synthesis, NP‐microbe synergism, and microbiome‐aware design, these nanosystems can deliver precision pathogen suppression while preserving or even enhancing beneficial microbial communities. The path forward requires coupling material innovation with microbial ecology, embedding SbD principles, and validating performance in multiseason, multisite trials that capture real‐world complexity. Regulatory harmonization, microbiome impact assessment, and life‐cycle analysis will be critical to ensure safety and societal trust. Looking ahead, convergence with synthetic biology and AI‐guided NP design could enable living microbial factories that produce antifungal NPs in situ, programmable nanobiofungicides that respond to pathogen signals, and intelligent coatings that self‐degrade after use. Such advances will transform antifungal nanotechnology into a cornerstone of sustainable microbial biotechnology, offering a scalable, ecologically responsible approach to fungal disease management in agriculture, food systems, and beyond.

## Author Contributions

**Kaveh Rahimi Mamaghani:** conceptualization, formal analysis, investigation, writing – original draft, data curation. **Marzieh Alikarami**: validation, writing – original draft, visualization. **Hossein Saremi**: resources, writing – review and editing, supervision, project administration, funding acquisition. **Nader Parvin**: writing – review and editing, supervision.

## Funding

The authors received no specific funding for this work.

## Conflicts of Interest

The authors declare no conflicts of interest.

## Supporting Information

Additional supporting information can be found online in the Supporting Information section.

## Supporting information


**Supporting Information** Table S1: Recent reviews on antifungal nanoparticles.

## Data Availability

No data were used for the research described in the article.
