# Supplementary material for: Antifungal Nanoparticles in Microbial Biotechnology: Multisector Mechanisms, Applications, and Future Frontiers
Source: IET Nanobiotechnol. 2026 May 30;2026:7866252. doi: 10.1049/nbt2/7866252 (PMC13239254; doi:10.1049/nbt2/7866252)
Supplement: Supplementary file 1 — Supporting Information Table S1: Recent reviews on antifungal nanoparticles. [file NBT2-2026-7866252-s001.docx]

**Antifungal Nanoparticles in Microbial Biotechnology: Multisector Mechanisms, Applications, and Future Frontiers**

Kaveh Rahimi Mamaghani^1,*^, Marzieh Alikarami^2^, Hossein Saremi^2,^*, Nader Parvin^1^

*^1^Department of Materials and Metallurgical Engineering, Amirkabir University of Technology (Tehran Polytechnic), Tehran, Iran*

*^2^Department of Plant Protection, College of Agriculture and Natural Resources, University of Tehran, Karaj, Iran*

*Corresponding authors. E-mail addresses:

[kaveh.metal85@gmail.com](mailto:kaveh.metal85@gmail.com), krahimi@aut.ac.ir (K.R. Mamagahni)

[hsn.saremi@ut.ac.ir](mailto:hsn.saremi@ut.ac.ir) (H. Saremi).

**Table S1.** Recent reviews on antifungal nanoparticles.

| **Reference** | **Scope of Review** | **Primary Focus** | **Emphasis on Microbiome /Agricultural Nano-Biofungicides** | **Regulatory / Safe-by-Design Discussion** | **Relevance to Current Manuscript** |
| --- | --- | --- | --- | --- | --- |
| Zhu *et al.* (2024) [1] | Antifungal nanoparticles in therapeutic (clinical) contexts | Biomedical antifungal nanomaterials, resistance issues | Mostly clinical, not microbiome/eco focus | No regulatory analysis | Establishes traditional focus on biomedical applications, highlighting need for broader biotechnology framework |
| Irshad *et al.* (2024)  [2] | Agricultural applications of green-synthesized NPs | Sustainable crop protection with metallic NPs | Discusses agricultural use, mechanisms, and sustainable strategies | Limited regulatory perspective | Represents agriculture-centric review but lacks biotechnology-regulation integration |
| Vijayalakshmi *et al.* (2025)  [3] | Antifungal properties of metal and metal-oxide NPs | Mechanisms of action and material properties | Focus on material and mechanism, not microbiome | General antimicrobial focus without regulatory depth | Good mechanistic review but does not integrate safe-by-design nor synthetic biology insights |
| This Review | Nano-biofungicides in microbial biotechnology | Integrated mechanisms, microbial ecology, microbiome, safe-by-design, regulatory gaps | Quantitative microbiome assessment; ecosystem perspective | Explicit regulatory and safe-by-design discussion | Synthesizes multiple aspects into a comprehensive biotechnology-oriented antifungal NP review |

**References:**

[1] X. Zhu, Y. Chen, D. Yu, W. Fang, W. Liao, W. Pan, Progress in the application of nanoparticles for the treatment of fungal infections: A review, Mycology, 15 (2024) 1-16.

[2] M.A. Irshad, A. Hussain, I. Nasim, R. Nawaz, A.A. Al-Mutairi, S. Azeem, M. Rizwan, S.A. Al-Hussain, A. Irfan, M.E.A. Zaki, Exploring the antifungal activities of green nanoparticles for sustainable agriculture: a research update, Chemical and Biological Technologies in Agriculture, 11 (2024) 133.

[3] K. Vijayalakshmi, T. Swaramanjari, M. Shanmugavel, A. Gnanamani, Green-synthesized metal and metal oxide nanoparticles as emerging antifungal agents: current advances, mechanisms, and future perspectives, Discover Biotechnology, 2 (2025) 18.
